# Examining the causal relationship between sex hormone-binding globulin (SHBG) and infertility: A Mendelian randomization study

**DOI:** 10.1371/journal.pone.0304216

**Published:** 2024-06-07

**Authors:** Hanghao Ma, Yan Chen

**Affiliations:** 1 Department of Internal Medicine, Ningde People’s Hospital, Ningde, Fujian, China; 2 Department of Ultrasound, Fujian Maternity and Child Health Hospital College of Clinical Medicine for Obstetrics & Gynecology and Pediatrics, Fujian Medical University, Fuzhou, China; Zhejiang University College of Life Sciences, CHINA

## Abstract

**Background:**

The causal relationship between sex hormone-binding globulin (SHBG) and infertility has remained unclear. Thus, we used Mendelian randomization (MR) to investigate this relationship.

**Methods:**

Risk factors for SHBG were extracted from European individuals within the UK Biobank using single-nucleotide polymorphism (SNP) data. Summary-level data for infertility outcomes were obtained from the FinnGen dataset. The causal relationship between SHBG and infertility was examined using inverse variance weighted, weighted model, weighted median, and MR-Egger regression analyses. Additionally, Cochran’s Q test and Egger intercept tests were used to confirm the heterogeneity and pleiotropy of identified instrumental variables (IVs).

**Results:**

Our findings revealed a significant negative association between sex hormone-binding globulin (SHBG) levels and infertility, particularly with anovulation, a specific form of female infertility. However, SHBG did not exert a causal impact on male infertility or on female infertility of tubal origin.

**Conclusions:**

SHBG expression offers protection against the development of certain types of female infertility, suggesting it is a potential therapeutic target for infertility.

## Introduction

Infertility is defined as unsuccessful pregnancy after 12 months of regular unprotected sex [1] and affect between 8 and 12% of reproductive-aged global couples [2]. While approximately 12.7% of American women of reproductive age seek treatment for infertility [3], the prevalence of infertility is steadily increasing worldwide and constrained by environmental changes and other related factors [4]. Infertility has multifaceted effects on the quality of life for couples, with unsuccessful family planning leading to emotional strain that often makes this phase the most stressful period in their lives [5]. thus, reproductive capability is closely associated with self-image and family harmony. Infertility can alter sexual behavior, affecting intimacy and potentially indirectly leading to sexual dysfunction, further complicating pregnancy [6]. Beyond the psychological toll on couples, infertility can also affect societal stability [7]; Given the escalating prevalence of infertility, identifying and treating modifiable infertility risk factors could serve as potential therapeutic strategies to improve family well-being and reduce the public health burden.

Sex hormone-binding globulin (SHBG) was first discovered by Mercier et al. (1966) [8] and is one the main proteins that bind to the circulating sex hormone alongside testosterone and estradiol [9]. The free hormone hypothesis states that SHBG is indispensable in the regulation of sex hormone bioavailability at target sites [10–12]. Currently, SHBG is primarily synthesized in the liver as a homodimeric glycoprotein, classified as a hepatokine, which can exert significant effects on ovarian function and lipid metabolism. To the best of our knowledge, a direct causal relationship between sex hormone-binding globulin (SHBG) and infertility has not been sufficiently established, primarily due to the lack of comprehensive and robust research. Therefore, we investigated the causal relationship between SHBG levels and infertility using a Mendelian randomization (MR) study.

Mendelian randomization is an analysis of genetic variables that follows the Mendelian law of inheritance and uses single nucleotide polymorphisms (SNPs) as instrumental variables (IVs) to infer observed causal relationships between modifiable exposure and clinically relevant outcomes. Alleles segregate randomly during meiosis, enabling MR to mitigate bias arising from confounding factors [13–15]. Randomized controlled clinical trials have substantial evidentiary value in determining causality; however, ethical and economic considerations limit their clinical implementation and make them difficult to perform. An MR approach can minimize the confounding effects of environmental exposure or socioeconomic status and obtain estimates to avoid potentially harmful interventions. It can also assess causality and reduce the influence of reverse causality [13, 15]. Thus, we used an MR analysis as a hypothesis-generating method to determine if there is a causal relationship between SHBG and infertility.

## Materials and methods

### Study design

In this study, SHBG was used as an exposure factor and infertility was designated as an outcome factor in the genome-wide association (GWAS). Two-sample MR was performed to evaluate the causal relationship between SHBG and infertility risk. Cochran’s Q test, Egger intercept tests and leave-one-out analysis were performed to determine the robustness of the MR estimates.

### Data source

Data on infertility obtained from the FinnGen Consortium R10 release (https://www.finngen.fi/) were used in this study. For male infertility, the patient and control groups comprised 1,429 and 130,139 cases, respectively. For female infertility, the patient and control groups comprised 14,759 and 111,583 cases, respectively. For female infertility associated with anovulation, the patient and control groups included 2,792 and 111583 cases, respectively. For tubal-origin infertility, the patient and control groups comprised 1,647 and 111,583 cases, respectively. The GWAS data for SHBG included 185,221 male cases and 214,989 female cases of European ancestry.(https://gwas.mrcieu.ac.uk/datasets). Table 1 provides a detailed description of these data.To avoid pleiotropic deviation of cross-lineage cases [15], all research participants were of European ancestry.

**Table 1:** Brief description of the genome-wide association study data used in this study.

GWAS ID	Year	Trait	sex	Consortium Sample size	Consortium Sample size Number of SNP
ieu-b-4870	2020	Sex hormone-binding globulin (SHBG)	female	214,989	12,321,875
ieu-b-4871	2020	Sex hormone-binding globulin (SHBG)	male	185,221	12,321,875
N14_FEMALEINFERT	2023	Female infertility	female	ncase 14759, ncontrol 111583	21,275,092
N14_MALEINFERT	2023	Male infertility	male	ncase 1429, ncontrol 130139	21,277,625
N14_FITUB	2023	Female infertility, tubal origin	female	ncase1647, ncontrol 111583	21,266,961
N14_FIANOV	2023	Female infertility, associated with anovulation	female	ncase2792, ncontrol 111583	21,267,799

### Instrumental variables

**Fig 1:**
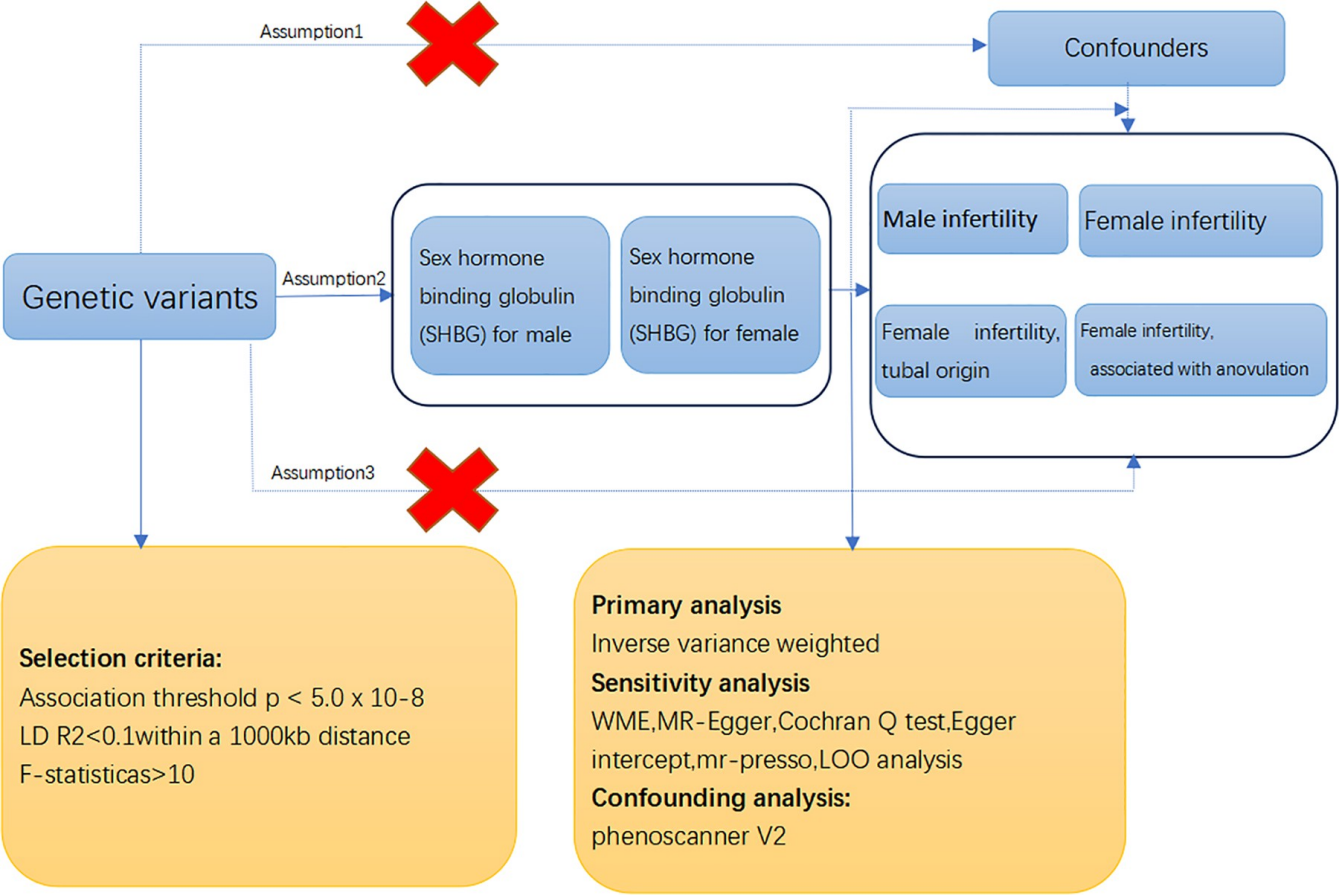
Design and workflow of the Mendelian randomization study of the causal relationship between sex hormone binding globulin (SHBG) and infertility. *IVW*, inverse variance weighted; *MR*, Mendelian randomization; *MR-PRESSO*, MR pleiotropy residual sum and outlier; *SNP*, single-nucleotide polymorphisms.

In this study, the criteria for selecting genetic variations as IVs were as follows: (1) SNPs were strongly associated with SHBG, (2) SNPs were independent of confounding factors that influenced both SHBG and infertility, and (3) SNPs were not directly related to infertility, with their effects on infertility mediated solely through SHBG. To identify optimal IVs related to SHBG characteristics, SNPs that were significantly related to male and female SHBG (*P* < 5.0×10^−8^) were identified (Fig 1).

The threshold value for R^2^ was set to 0.001 and the kilobase pair (KB) was set to 10,000 to eliminate interference from linkage disequilibrium (LD). SNPs that could not be used to identify alternative sites were eliminated. The F-statistics were used to evaluate weak IV bias, with F > 10 indicating a strong IV. The calculation formula is:

$$F=\left[\frac{N-k-1}{K}\right]*[\frac{R2}{1-R2}]$$ (1)

where *n* is the sample size of the exposure database, *K* is the number of SNPs, and *R*^*2*^ is the proportion of variation explained by the SNPs in the exposure database. The formula for calculating R^2^ is:

$$R2=[2*EAF*(1-EAF)*\beta^{2}]$$ (2)

where *EAF* is the effective allele frequency, *β* is the allelic effect value. We also checked in PhenoScanner (www.phenoscanner.medschl.cam.ac.uk), a platform with comprehensive information on the association of genotype and phenotype, to confirm these SNPs were associated with the potential risk factors, and remove SNPs associated with any potential confounders with genome-wide significance.

### MR analysis

Four different methods were employed for MR analysis: (1) the inverse variance weighted (IVW) method, (2) MR-Egger regression, (3) weighted median, and (4)weighted model. (1) We primarily used the IVW method to validate causal relationships. When there is no observed variation in the experiment, the IVW results are considered unbiased. This key concept involves assigning weights to the results of each study based on the study’s variance [16]. IVW can avoid confounding variables without a horizontal pleiotropy and produce unbiased estimation. In the absence of horizontal pleiotropy, the statistical effect of IVW is significantly stronger than the other three methods. (2) The MR-Egger regression was based on the assumption of instrument strength independent of direct effect (InSIDE) and considers the potential presence of pleiotropy. It accordingly adjusts the results to provide more accurate estimates. If there is evidence of pleiotropy, the MR-Egger regression is used to further analyze and improve accuracy [17]. (3) The weighted median method uses the median to mitigate the potential influence of outliers in genetic instruments. Moreover, this method can correctly estimate the causal association even when up to 50% of IVs are invalid. Therefore, when most IVs are ineffective, this method is used to assess causal relationships [18, 19]. (4) Notably, weighted model estimation can outperform the MR-Egger regression in situations where the InSIDE hypothesis is violated because it demonstrates greater statistical power for detecting causal effects, reduced bias, and lower rates of type I errors [19, 20].

The MR Pleiotropy RESidual Sum and Outlier (MRPRESSO) tests are optimally applicable when a horizontal pleiotropy is found in < 50% of the instruments [21] and were applied to remove the underlying outliers before each MR analysis. Although MR is more effective than observational studies in avoiding confounding biases, we conducted sensitivity analyses to ensure the reliability of our MR estimates.

Cochran’s Q test is a statistical test used to determine if there are significant differences between multiple dependent variables within a single group, based on categorical data. We used Cochran’s Q test to evaluate heterogeneity, where a *P*-value > 0.05 indicated the absence of heterogeneity.

An Egger intercept analysis was used to evaluate horizontal pleiotropy. An Egger intercept is a statistical term referring to the intercept of the regression line in Egger’s regression test, which is used to assess publication bias in meta-analysis studies. This analysis identifies the presence of small-study effects or asymmetry in a funnel plot, suggesting potential bias in the literature.

Leave-one-out analysis evaluates the effect of an observation on sample data by excluding the overall result [22].

### Confounding analysis

An array of statistical methods were conducted in sensitivity analysis to assess any violation of the MR assumptions. Additionally, we utilized the Phenoscanner V2 website (http://www.phenoscanner.medschl.cam.ac.uk/) to explore whether the infertility-associated SNPs were concurrently associated with several common risk factors that could potentially bias the MR estimate. For women, these factors included body mass index (BMI) [22] and polycystic ovary syndrome (PCOS) [23], whereas for men, BMI was considered [24]. Subsequently, if the SNPs were found to be associated with these potential confounders at a threshold of P < 5 × 10^−8^, IVW was replicated after removing these SNPs to validate the robustness of the results.

### Statistical analyses

All analyses were performed using the packages TwoSampleMR (version 0.5.7) in R (version 4.2.1). *P* < 0.05 was used to indicate significance.

### Ethical statement

This study used publicly available de-identified data from participant studies that had obtained approved by ethical standards committees with respect to human experimentation. No separate ethical approval was required in this study.

## Results

### Instrument variables for SHBG

After removing the IVs with linkage disequilibrium, we identified 197 SNPs associated with SHBG expression in women and 200 SNPs s associated with SHBG in men (SNP information is listed in S1 and S2 Tables). Given the established association between diabetes and infertility [23], seven SNPs (rs663129, rs34835, rs58939796, rs62011286, rs7947951, rs511154, and rs9379802) associated with BMI were considered potential confounding factors and accordingly excluded from the analysis for men. Similarly, for women, nine SNPs (rs1014291, rs13086465, rs687339, rs998584, rs7947951, rs62033400, rs12444108, rs663640, rs273492) associated with BMI were considered potential confounding factors for infertility outcomes and excluded from the analysis. No other potential confounding factors of infertility, such as PCOS and sex hormones, were identified. Moreover, MR-PRESSO analysis revealed no outliers in any of the results.

### MR analysis of SHBG and female infertility

The IVW analysis demonstrated a causal relationship between SHBG and female infertility (OR = 0.906, 95% CI = 0.837–0.98, P = 0.014). Similar risk estimates were obtained using MR-Egger (OR = 0.864, 95% CI = 0.753–0.991, P = 0.039), weighted median (OR = 0.844, 95% CI = 0.752–0.947, P = 0.004), and weighted mode estimate (OR = 0.832, 95% CI = 0.734–0.943, P = 0.005) analyses, all of which were statistically significant, indicating that SHBG levels were associated with female infertility (Table 2).

**Table 2:** MR results on sex hormone binding globulin (SHBG) and infertility.

Outcome	Method	nsnp	beta	se	pval	or	or_lci95	or_uci95
Female infertility	MR Egger	169	-0.146	0.07	0.039	0.864	0.753	0.991
	Weighted median	169	-0.17	0.059	0.004	0.844	0.752	0.947
	Inverse variance weighted	169	-0.099	0.04	0.014	0.906	0.837	0.98
	Weighted mode	169	-0.184	0.064	0.005	0.832	0.734	0.943
Female infertility associated with anovulation	MR Egger	169	-0.379	0.134	0.005	0.685	0.527	0.89
	Weighted median	169	-0.269	0.124	0.03	0.764	0.6	0.974
	Inverse variance weighted	169	-0.289	0.077	1.67E-04	0.749	0.645	0.871
	Weighted mode	169	-0.386	0.121	0.002	0.68	0.537	0.861
Female infertility with a tubal origin	MR Egger	169	-0.274	0.174	0.117	0.76	0.54	1.07
	Weighted median	169	-0.175	0.156	0.264	0.84	0.618	1.141
	Inverse variance weighted	169	-0.154	0.1	0.123	0.857	0.705	1.043
	Weighted mode	169	-0.211	0.168	0.21	0.81	0.583	1.125
Male infertility	MR Egger	179	-0.066	0.173	0.703	0.936	0.667	1.314
	Weighted median	179	0.029	0.145	0.841	1.03	0.775	1.369
	Inverse variance weighted	179	-0.047	0.098	0.627	0.954	0.788	1.155
	Weighted mode	179	-0.092	0.158	0.56	0.912	0.669	1.243

*Note*: *p* < 0.05 represents the significance of the causal association of decreased SHBG and infertility. Abbreviations: *β*, the regression coefficient based on the sex hormone binding globulin raising effect allele; IVW, inverse variance weighted; MR, Mendelian randomization; nsnp, number of single-nucleotide polymorphism; OR, odds ratio; OR_lci95, lower limit of 95% confidence interval for OR; OR_uci95, upper limit of 95% confidence interval for OR; SE, standard error.

**Fig 2:**
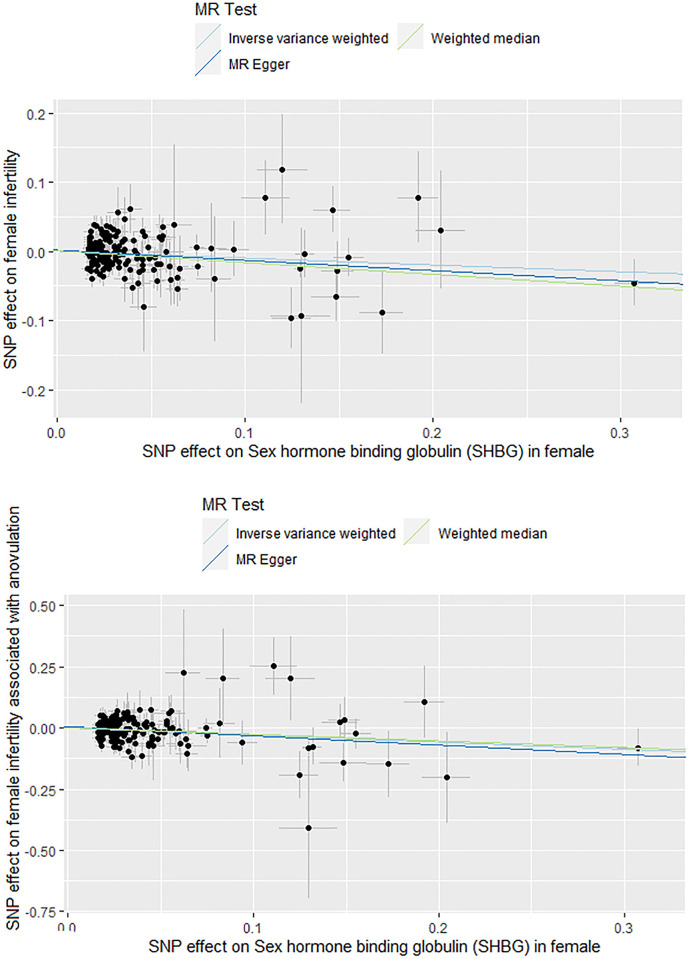
Funnel plots illustrating the causal relationship between sex hormone binding globulin (SHBG) and infertility. (A) Funnel plot for the causal association between SHBG and female infertility (B). Funnel plot for the causal association between SHBG and Female infertility associated with anovulation.

**Fig 3:**
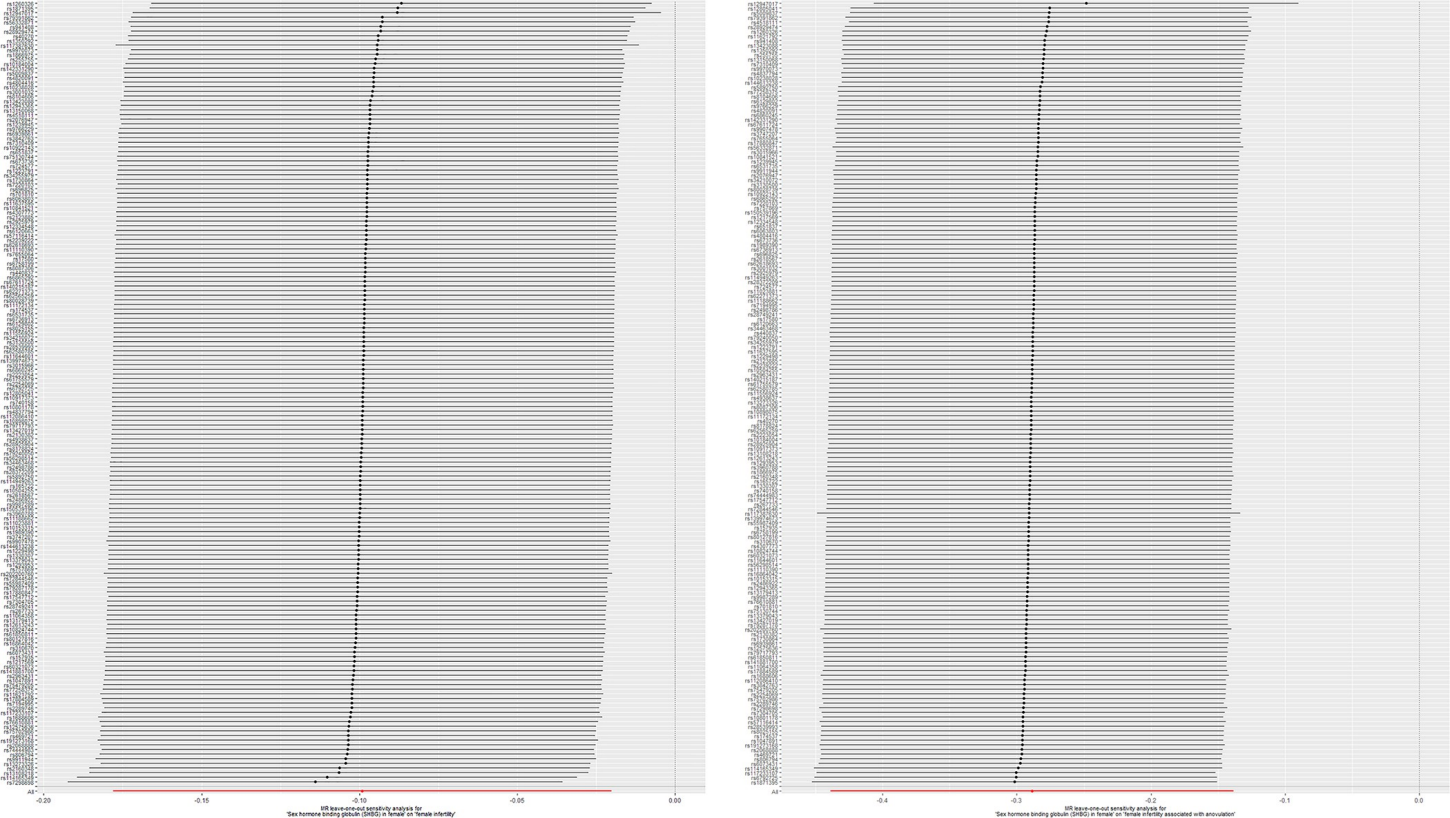
Leave-one-out plots for the causal relationship between sex hormone binding globulin (SHBG) and infertility. (A) Leave-one-out plots for the causal relationship between SHBG and female infertility. (B) Leave-one-out plots for the causal relationships between SHBG and female infertility associated with anovulation.

Horizontal pleiotropy was not detected in the MR-Egger intercept (P = 0.414; Table 4), and Egger intercepts did not identify any pleiotropy, suggesting the absence of pleiotropicin in the MR estimates within the context of heterogeneity. Moreover, Cochran’s Q-test detected heterogeneity (P = 1.17×10^−05^; Table 3). Despite this heterogeneity, MR analysis did not introduce pleiotropic bias, and the IVW method balanced the pooled heterogeneity, ensuring the validity of the MR assessment as a random-effects model. The scatter plots illustrating these findings are presented in Fig 2A. Moreover, the results of the leave-one-out analysis indicated that no SNP influenced the IVW results (Fig 3A).

**Table 3:** Cochran’s Q test results on sex hormone binding globulin (SHBG) and infertility.

Outcome	MR analysis	Q	Global P value
Female infertility	MR Egger	255.996	1.13×10^−05^
	Inverse variance weighted	257.025	1.17×10^−05^
Female infertility, associated with anovulation	MR Egger	191.859	0.091
	Inverse variance weighted	192.638	0.094
Female infertility, tubal origin	MR Egger	196.461	0.059
	Inverse variance weighted	197.298	0.061
Male infertility	MR Egger	228.294	0.006
	Inverse variance weighted	228.315	0.006

### MR analysis of SHBG and female infertility associated with anovulation

The IVW analysis demonstrated a negative causal link between SHBG and female infertility associated with anovulation (OR = 0.749, 95% CI0.645–0.871, P = 1.67×10^−4^). The effects of SHBG on infertility were also assessed using the IVW method, and consistent estimates were obtained using the Weighted median method (OR = 0.764, 95% CI = 0.6–0.974, P = 0.03). However, two negative and insignificant estimates were observed in the MR-Egger (OR = 0.685, 95% CI = 0.527–0.89, P = 0.005) and weighted mode (OR = 0.68, 95% CI0.537–0.861, P = 0.002) analyses (Table 2). Together, these results indicate that SHBG may contribute to reduced female infertility associated with anovulation.

Cochran’s Q-test revealed no heterogeneity (P = 0.094), and the MR-Egger intercept analysis indicated no horizontal pleiotropy, providing strong evidence of the absence of heterogeneity and pleiotropy (P = 0.400; Tables 3 and 4). The results of the leave-one-out analysis and MR-PRESSSO indicated that none of the SNPs significantly affected the IVW results. Furthermore, the results of the leave-one-out analysis suggested that the observed association did not change significantly after removing any single variant. “Funnel plots illustrating these findings are presented in Fig 2. Moreover, the leave-one-out analysis results corroborate that individual SNPs did not introduce bias in the MR estimation (Fig 3B).

**Table 4:** Egger intercept analysis of sex hormone binding globulin (SHBG) and infertility.

Outcome	egger_intercept	se	pval
Female infertility	0.002	0.003	0.414
Female infertility associated with anovulation	0.005	0.006	0.400
Female infertility tubal origin	0.004	0.005	0.412
Male infertility	0.001	0.007	0.897

### MR analysis of SHBG and female infertility with a tubal origin

Overall, SHBG was significantly associated with female infertility. However, the IVW analysis revealed no causal relationship between SHBG levels and female infertility with a tubal origin (OR = 0.857, 95% CI = 0.705–1.043, P = 0.123). Consistent estimates were obtained through MR-Egger (OR = 0.76, 95% CI0.54–1.07, P = 0.117), weighted median (OR = 0.84, 95% CI = 0.618–1.141, P = 0.264), and weighted mode (OR = 0.81, 95% CI = 0.583–1.125, P = 0.21) methods, which were not statistically significant (Table 2). Cochran’s Q-test revealed no heterogeneity (*P* = 0.061), and horizontal pleiotropy was absent in the MR-Egger intercept analysis (P = 0.412; Tables 3 and 4).

### MR analysis of SHBG and male infertility

The IVW analysis indicated no causal relationship between SHBG and male infertility(OR = 0.954, 95% CI = 0.788–1.155, P = 0.627) Similar results were obtained in the MR-Egger (OR = 0.936, 95% CI = 0.667–1.314, P = 0.703) and weighted median (OR = 1.03, 95% CI = 0.775–1.369, P = 0.841) analyses, as well as the weighted mode method (OR = 0.912, 95% CI = 0.669–1.243, P = 0.56), and they were not significant (Table 2). Cochran’s Q test indicated the presence of heterogeneity (P = 0.006), but the IVW effectively balanced this heterogeneity (Table 3). Moreover, the MR-Egger intercept analysis indicated no evidence of horizontal pleiotropy (P = 0.897; Table 4).

## Discussion

Currently, the causal relationship between SHBG and female infertility remains unclear. To our knowledge, this study is the first to investigate the genetic causal associations between SHBG and infertility, thereby making a significant contribution to the understanding of infertility mechanisms. Establishing a causal relationship between SHBG levels and infertility holds significant implications for those affected by infertility. In this study, we used five sets of IVs to classify infertility: (1) female infertility; (2) female infertility due to anovulation; (3) female infertility due to a tubal origin; and (4) male infertility. Using VWI-based MR analysis, we found that SHBG was negatively correlated with female infertility. Among the different types of infertility tested, SHBG was negatively correlated with specific categories, such as infertility associated with anovulation. However, SHBG showed no causal effect relationship on male or female infertility with a tubal origin.

Our study had the following advantages: (1) It underscored the significant sex-based disparities in the correlation between SHBG and infertility, with a distinct categorization based on sex. (2) The MR analysis method mitigated the impact of confounding factors, reverse causality, and reporting bias to ensure a comprehensive assessment of the risk associations caused by phenotypic and behavioral variation in the data. (3) Large-scale GWAS datasets for SHBG and infertility were used in this study, which enhanced the strength of the statistical analysis, especially when analyzing the effects of SHBG on disease outcomes.

**Fig 4:**
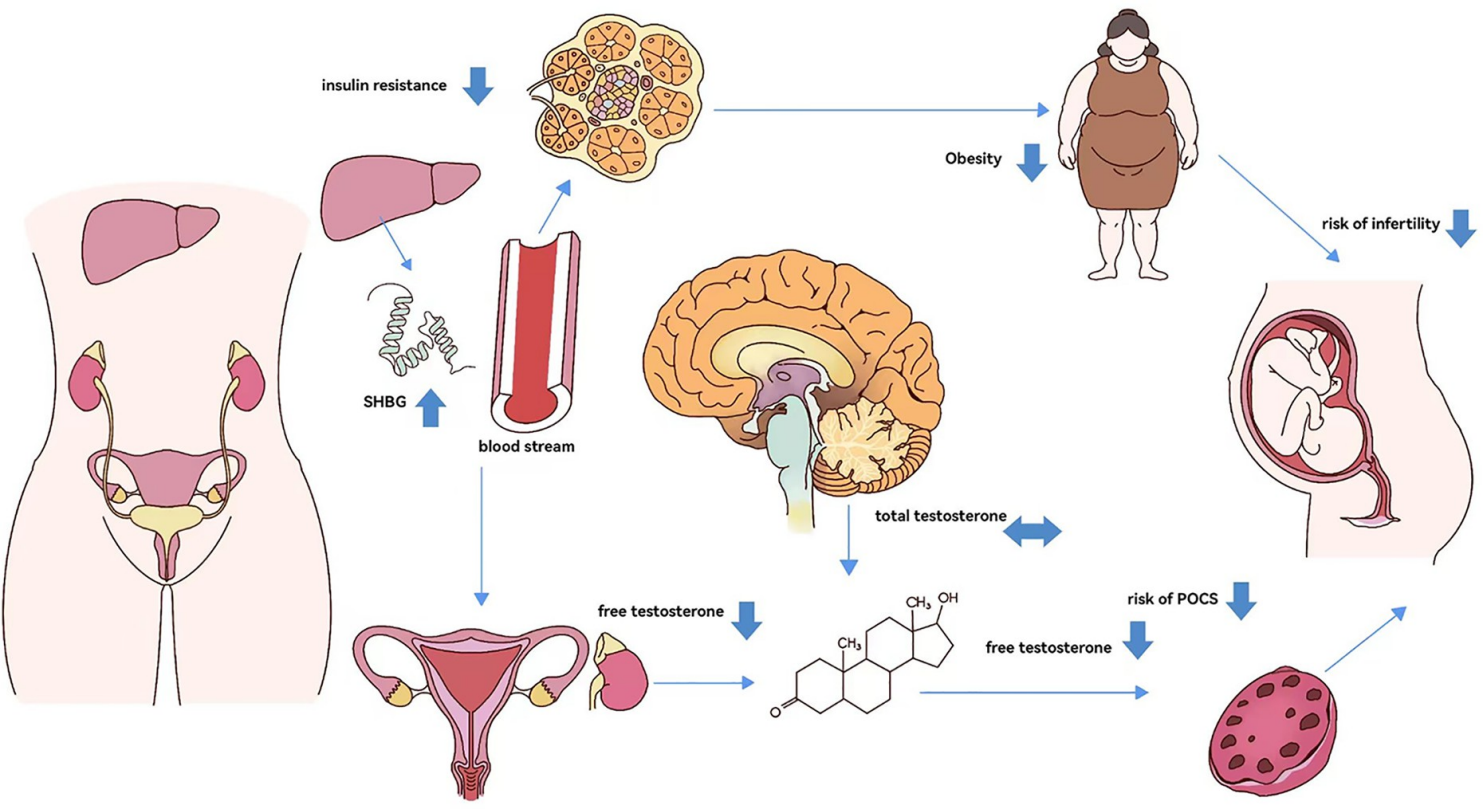
Potential mechanisms by which SHBG may cause infertility.

Zhai et al. reported a positive correlation between serum SHBG concentrations and ovarian response in patients without PCOS during ovarian hyperstimulation [24]. However, the study did not provide additional insights into the relationship between serum SHBG concentration and pregnancy outcomes. Moreover, Gunning et al. demonstrated that high SHBG levels in oligo/anovulatory women with PCOS were associated with full-term live births, suggesting that SHBG could increase the chances of successful pregnancy in anovulatory women. The study further confirmed that SHBG serves as a protective factor in anovulatory infertility in women [25]. Previous research has predominantly focused on the relationship between SHBG and ovarian dysfunction or metabolic disorders in patients with PCOS without further analyzing SHBG as a protective factor in female patients with infertility [25–27]. According to existing research, SHBG acts as a protective factor in female infertility through various mechanisms; lower SHBG levels result in elevated free testosterone and insulin resistance, increasing the likelihood of obesity and PCOS, and consequently, the risk of infertility. Therefore, SHBG could prevent the occurrence and development of infertility by jointly acting through these two mechanisms [26, 27]. In insulin-resistant cells, reduced mRNA and protein levels of SHBG are correlated with decreased expression levels of IRS-1, IRS-2, PI3Kp85a, GLUT-3, and GLUT-4 [28, 29]. This indicates that low SHBG expression may suppress the PI3K/AKT pathway, thereby contributing to the development of insulin resistance [9, 28]. This finding, combined with our results, suggests that low SHBG levels are associated with a high risk of infertility, further confirming that adequate SHBG expression can improve infertility caused by anovulation associated with ovarian dysfunction, which is consistent with previous indirect evidence [24–28]. In women, serum SHBG levels are negatively correlated with free testosterone levels, suggesting that SHBG does not participate in a feedback loop through the hypothalamic–pituitary–gonadal axis. Thus, total testosterone levels, which are independently regulated by the ovaries and adrenal glands, remain unaffected. Additionally, elevated SHBG levels lead to a significant reduction in free testosterone levels. However, paradoxically, there is also a continuous increase in free circulating androgens, likely indicative of the involvement of the liver axis. This suggests that a reduction in SHBG synthesis in the liver could lead to insulin resistance and decreased androgen bioavailability [9]. Moreover, in patients with PCOS, obesity is associated with an increase in pro-inflammatory cytokines, insulin resistance, and apoptosis of ovarian granulosa cells [30]. These combined factors ultimately lead to hyperandrogenemia, metabolic disorders, and ovarian dysfunction, which can result in anovulation and infertility (Fig 4) [31–33].

We believe that our findings highlight the potential of SHBG as a therapeutic target. Some reports indicate that treatment with metformin, myo-inositol, insulin sensitizers, low-carbohydrate diets, and D-chiro-inositol can increase serum SHBG levels, improving ovarian ovulation function in patients with PCOS [34–40]. Peroxisome proliferator-activated receptor-γ (PPAR-γ) competes with HNF-4α for binding at the DR3 site on the *SHBG* promoter and acts as an inhibitor of *SHBG* expression. Decreasing the activity of PPAR-γ can, therefore, increase SHBG production [34]. However, some study reported that thiazolidinediones (i.e., PPAR-γ agonists) led to an increase in plasma SHBG levels and improved anovulation and ovulatory dysfunction and infertility in patients with PCOS [34, 40]. Combined with previous studies, our findings further verify that, for women in general, boosting SHBG levels with medication not only improves the progression of ovarian pathologies but also increases the probability of pregnancy. Therefore, serum SHBG levels could serve as useful biomarker for the diagnosis and potential therapeutic target infertility [34–40].

Although we used MR analysis, the study had some limitations. The population included in the study was mainly of European descent. Because the results of the causal association analysis could be affected by race, we would need to conduct the same MR study using data from populations that included different races to verify this conclusion.

This Mendelian Randomization (MR) study revealed that higher circulating levels of sex hormone-binding globulin (SHBG) are linked to a reduced risk of female infertility, particularly in cases of anovulation.Specifically, SHBG levels were found to be inversely associated with various types of female infertility, notably with anovulation.The SHBG levels were not related to male infertility or tubal-origin female infertility. The results of the two-sample MR analysis showed that SHBG is a protective factor against the development of infertility and could be used as a potential biomarker and therapeutic target for infertility.

## Supporting information

S1 Table Basic information of single nucleotide polymorphisms associated with sex hormone-binding globulin in female samples. (DOCX)

S2 Table Basic information of single nucleotide polymorphisms associated with sex hormone-binding globulin in male samples. (DOCX)
