# Supplementary material for: Examining the causal relationship between sex hormone-binding globulin (SHBG) and infertility: A Mendelian randomization study
Source: PLoS One. 2024 Jun 7;19(6):e0304216. doi: 10.1371/journal.pone.0304216 (PMC11161117; doi:10.1371/journal.pone.0304216)
Supplement: S1 Table — (DOCX) [file pone.0304216.s001.docx]

# Supplementary Table 1 Basic information of SNPs associated with sex hormone binding globulin in female

| SNP | EA | OA | eaf | beta | se | pval | r2 | f |
| --- | --- | --- | --- | --- | --- | --- | --- | --- |
| rs35504625 | G | C | 0.476383 | 0.0299822 | 0.0029524 | 3.10E-24 | 4.48E-04 | 96.4570534 |
| rs144613238 | A | AATTT | 0.924926 | -0.0345806 | 0.00561056 | 7.10E-10 | 1.66E-04 | 35.70885435 |
| rs28749241 | C | T | 0.498342 | -0.0169493 | 0.00295465 | 9.70E-09 | 1.44E-04 | 30.88469717 |
| rs9970073 | A | G | 0.361335 | 0.0181328 | 0.00307794 | 3.80E-09 | 1.52E-04 | 32.63029474 |
| rs3001032 | C | T | 0.318667 | 0.0209467 | 0.00316475 | 3.60E-11 | 1.91E-04 | 40.96874777 |
| rs469721 | T | C | 0.196625 | -0.0259489 | 0.00372693 | 3.30E-12 | 2.13E-04 | 45.74351622 |
| rs10922143 | A | G | 0.66436 | 0.0241053 | 0.00312809 | 1.30E-14 | 2.59E-04 | 55.7258912 |
| rs1223791 | A | G | 0.836612 | -0.0289385 | 0.00399386 | 4.30E-13 | 2.29E-04 | 49.23091567 |
| rs2486922 | C | T | 0.74269 | -0.023057 | 0.0033718 | 8.00E-12 | 2.03E-04 | 43.69183533 |
| rs114165349 | C | G | 0.023317 | -0.146504 | 0.00984242 | 4.10E-50 | 9.78E-04 | 210.3740798 |
| rs12121301 | A | T | 0.436024 | 0.0187813 | 0.00297553 | 2.80E-10 | 1.73E-04 | 37.30266508 |
| rs1730864 | T | C | 0.659135 | -0.0425018 | 0.00313228 | 6.10E-42 | 8.12E-04 | 174.649075 |
| rs267733 | G | A | 0.160766 | -0.0272953 | 0.00400964 | 9.90E-12 | 2.01E-04 | 43.22973118 |
| rs16864042 | T | G | 0.415 | -0.0173313 | 0.00299143 | 6.90E-09 | 1.46E-04 | 31.35969157 |
| rs10917373 | T | C | 0.74479 | 0.0224874 | 0.00339598 | 3.50E-11 | 1.92E-04 | 41.33669075 |
| rs201468966 | C | CA | 0.767149 | 0.0266675 | 0.00351214 | 3.10E-14 | 2.54E-04 | 54.63550835 |
| rs36209093 | T | C | 0.673522 | -0.0250211 | 0.00350159 | 9.00E-13 | 2.75E-04 | 59.20798609 |
| rs10801178 | C | G | 0.689629 | 0.0185299 | 0.00318804 | 6.20E-09 | 1.47E-04 | 31.60449369 |
| rs1260326 | C | T | 0.605329 | 0.0637846 | 0.00301669 | 3.10E-99 | 1.94E-03 | 418.7411479 |
| rs12613243 | C | T | 0.063353 | -0.0355853 | 0.00606463 | 4.40E-09 | 1.50E-04 | 32.31408495 |
| rs13427019 | A | T | 0.237235 | -0.0195333 | 0.00348208 | 2.00E-08 | 1.38E-04 | 29.69087037 |
| rs1047891 | A | C | 0.315465 | 0.0256461 | 0.00316928 | 5.90E-16 | 2.84E-04 | 61.08788435 |
| rs6758199 | T | C | 0.06703 | -0.0454102 | 0.00595386 | 2.40E-14 | 2.58E-04 | 55.4623092 |
| rs6736913 | G | A | 0.979622 | -0.0649187 | 0.0104228 | 4.70E-10 | 1.68E-04 | 36.18053578 |
| rs1014291 | T | G | 0.427368 | 0.0178266 | 0.00298929 | 2.50E-09 | 1.56E-04 | 33.44447666 |
| rs10184004 | T | C | 0.405686 | 0.0330313 | 0.00300414 | 4.00E-28 | 5.26E-04 | 113.1691521 |
| rs2160348 | C | T | 0.735302 | -0.0560969 | 0.00336141 | 1.60E-62 | 1.22E-03 | 263.6748142 |
| rs13423088 | A | T | 0.316906 | 0.0290991 | 0.00317326 | 4.70E-20 | 3.67E-04 | 78.84451498 |
| rs4518111 | C | A | 0.566236 | 0.0258866 | 0.0029982 | 5.90E-18 | 3.29E-04 | 70.79232689 |
| rs62271373 | A | T | 0.060056 | -0.0456189 | 0.00634288 | 6.40E-13 | 2.35E-04 | 50.52336243 |
| rs79287178 | A | G | 0.030823 | -0.0543203 | 0.00897452 | 1.40E-09 | 1.76E-04 | 37.90714613 |
| rs6792725 | G | A | 0.69137 | 0.0349923 | 0.00329631 | 2.50E-26 | 5.23E-04 | 112.3991017 |
| rs13086465 | T | C | 0.320168 | -0.0277625 | 0.00316041 | 1.60E-18 | 3.36E-04 | 72.15801974 |
| rs2289746 | C | T | 0.66807 | 0.0176246 | 0.00314665 | 2.10E-08 | 1.38E-04 | 29.62163621 |
| rs56298514 | C | A | 0.240339 | -0.0198062 | 0.00346149 | 1.10E-08 | 1.43E-04 | 30.80000668 |
| rs687339 | T | C | 0.772208 | -0.0683786 | 0.00352857 | 1.20E-83 | 1.64E-03 | 354.2177613 |
| rs724577 | C | A | 0.73841 | -0.019965 | 0.00335904 | 2.80E-09 | 1.54E-04 | 33.11055138 |
| rs3960788 | C | T | 0.413016 | 0.0174139 | 0.00300405 | 6.80E-09 | 1.47E-04 | 31.61486179 |
| rs28925904 | T | C | 0.025113 | -0.0621911 | 0.00943409 | 4.30E-11 | 1.89E-04 | 40.72244105 |
| rs6531735 | A | G | 0.507025 | -0.0173749 | 0.00295808 | 4.30E-09 | 1.51E-04 | 32.44939753 |
| rs2076947 | C | T | 0.796968 | -0.0257823 | 0.00374068 | 5.50E-12 | 2.15E-04 | 46.25773052 |
| rs2123885 | G | T | 0.235023 | 0.0210116 | 0.00352624 | 2.50E-09 | 1.59E-04 | 34.13407482 |
| rs7655064 | C | T | 0.12535 | -0.0253791 | 0.00446946 | 1.40E-08 | 1.41E-04 | 30.36788944 |
| rs76610881 | G | A | 0.108666 | 0.0451543 | 0.0047491 | 1.90E-21 | 3.95E-04 | 84.94662794 |
| rs13108218 | G | A | 0.614831 | -0.0467657 | 0.00305625 | 7.50E-53 | 1.04E-03 | 222.9226764 |
| rs1866975 | T | C | 0.386973 | -0.022345 | 0.00304361 | 2.10E-13 | 2.37E-04 | 50.94083666 |
| rs13150068 | G | A | 0.435452 | -0.0329623 | 0.00298399 | 2.30E-28 | 5.34E-04 | 114.9080452 |
| rs17547712 | A | G | 0.078552 | 0.0321779 | 0.00553384 | 6.10E-09 | 1.50E-04 | 32.2292924 |
| rs6860245 | C | G | 0.247648 | 0.0252273 | 0.00343589 | 2.10E-13 | 2.37E-04 | 50.99676763 |
| rs6865292 | C | T | 0.2754 | -0.0187035 | 0.00337265 | 2.90E-08 | 1.40E-04 | 30.02001244 |
| rs2963431 | T | C | 0.237974 | -0.023049 | 0.0034808 | 3.50E-11 | 1.93E-04 | 41.43137611 |
| rs255755 | G | A | 0.269516 | -0.0245159 | 0.00335065 | 2.50E-13 | 2.37E-04 | 50.89040759 |
| rs13179413 | T | C | 0.283791 | -0.0228055 | 0.00333958 | 8.60E-12 | 2.11E-04 | 45.46232192 |
| rs40270 | C | A | 0.772585 | -0.038852 | 0.00352312 | 2.80E-28 | 5.30E-04 | 114.0945119 |
| rs67570751 | C | CT | 0.347295 | 0.0225895 | 0.00311022 | 3.80E-13 | 2.31E-04 | 49.74751427 |
| rs806794 | G | A | 0.275077 | 0.0239671 | 0.00330704 | 4.30E-13 | 2.29E-04 | 49.2627706 |
| rs3130500 | A | T | 0.743867 | -0.0269001 | 0.00338267 | 1.80E-15 | 2.76E-04 | 59.29670778 |
| rs9766229 | C | A | 0.041334 | 0.0433795 | 0.0075195 | 8.00E-09 | 1.49E-04 | 32.06644474 |
| rs6939861 | A | G | 0.262046 | -0.0374876 | 0.00340731 | 3.70E-28 | 5.44E-04 | 116.9123542 |
| rs673736 | A | G | 0.482874 | 0.0307208 | 0.00296955 | 4.40E-25 | 4.71E-04 | 101.37764 |
| rs998584 | A | C | 0.483837 | -0.0211222 | 0.00296727 | 1.10E-12 | 2.23E-04 | 47.91850189 |
| rs1293953 | C | G | 0.302711 | 0.0214646 | 0.00323468 | 3.20E-11 | 1.94E-04 | 41.82281773 |
| rs75479205 | G | A | 0.189884 | 0.0254311 | 0.00378104 | 1.70E-11 | 1.99E-04 | 42.78529095 |
| rs651837 | G | A | 0.590551 | 0.0195602 | 0.0030061 | 7.70E-11 | 1.85E-04 | 39.78564147 |
| rs740158 | C | T | 0.487513 | 0.0162306 | 0.00296785 | 4.50E-08 | 1.32E-04 | 28.30333233 |
| rs114949263 | C | T | 0.111485 | 0.0304011 | 0.00471238 | 1.10E-10 | 1.83E-04 | 39.37146001 |
| rs17884589 | A | G | 0.183948 | 0.0355752 | 0.00380609 | 9.00E-21 | 3.80E-04 | 81.7174574 |
| rs1229498 | G | T | 0.723431 | -0.0215258 | 0.00335963 | 1.50E-10 | 1.85E-04 | 39.86960602 |
| rs11556924 | T | C | 0.388304 | 0.023141 | 0.00303607 | 2.50E-14 | 2.54E-04 | 54.70467464 |
| rs157935 | G | T | 0.302551 | 0.0179322 | 0.00322814 | 2.80E-08 | 1.36E-04 | 29.17959994 |
| rs10622246 | A | ATTTT | 0.499827 | -0.0168973 | 0.00296184 | 1.20E-08 | 1.43E-04 | 30.6957879 |
| rs1688606 | A | G | 0.81594 | 0.0742507 | 0.00381577 | 2.40E-84 | 1.66E-03 | 356.5994838 |
| rs10238028 | G | A | 0.067698 | 0.0396673 | 0.00590167 | 1.80E-11 | 1.99E-04 | 42.70966845 |
| rs77258375 | C | CT | 0.638849 | -0.0258712 | 0.00311175 | 9.30E-17 | 3.09E-04 | 66.41964288 |
| rs12334548 | C | G | 0.078821 | -0.0359285 | 0.00548636 | 5.80E-11 | 1.87E-04 | 40.30767555 |
| rs5889386 | T | A | 0.387201 | -0.0178382 | 0.00326265 | 4.60E-08 | 1.51E-04 | 32.46865929 |
| rs2223054 | C | T | 0.570854 | -0.0217112 | 0.00299089 | 3.90E-13 | 2.31E-04 | 49.66383847 |
| rs9987289 | G | A | 0.9084 | 0.0409459 | 0.00512214 | 1.30E-15 | 2.79E-04 | 60.00077053 |
| rs10504255 | A | G | 0.663223 | 0.0195318 | 0.00313626 | 4.70E-10 | 1.70E-04 | 36.64398976 |
| rs150539196 | G | A | 0.035588 | 0.0940289 | 0.00822247 | 2.80E-30 | 6.07E-04 | 130.5553724 |
| rs5892750 | CA | C | 0.800246 | 0.0327869 | 0.00369428 | 7.00E-19 | 3.44E-04 | 73.91135878 |
| rs440837 | G | A | 0.20749 | 0.0519732 | 0.00364977 | 5.20E-46 | 8.88E-04 | 191.1564753 |
| rs13273326 | G | A | 0.530454 | -0.0192839 | 0.00297287 | 8.80E-11 | 1.85E-04 | 39.83256455 |
| rs62580785 | C | T | 0.1793 | 0.0224057 | 0.00387333 | 7.30E-09 | 1.48E-04 | 31.76787176 |
| rs4837794 | C | T | 0.668607 | -0.0259469 | 0.00315623 | 2.00E-16 | 2.98E-04 | 64.15891173 |
| rs112086410 | C | G | 0.125469 | -0.0301626 | 0.00448813 | 1.80E-11 | 2.00E-04 | 42.93170214 |
| rs7039093 | G | C | 0.424603 | 0.0184145 | 0.00299864 | 8.20E-10 | 1.66E-04 | 35.62744464 |
| rs28372209 | G | A | 0.525651 | -0.0164395 | 0.00297218 | 3.20E-08 | 1.35E-04 | 28.97833466 |
| rs2986670 | A | C | 0.324479 | -0.017205 | 0.00315234 | 4.80E-08 | 1.30E-04 | 27.90187311 |
| rs34463468 | T | C | 0.779051 | -0.0310248 | 0.00357781 | 4.30E-18 | 3.31E-04 | 71.2626617 |
| rs1330307 | C | A | 0.486123 | -0.0174195 | 0.00297209 | 4.60E-09 | 1.52E-04 | 32.59753497 |
| rs696825 | T | C | 0.253119 | 0.0488535 | 0.00340666 | 1.20E-46 | 9.02E-04 | 194.1787951 |
| rs62565259 | T | C | 0.171696 | 0.023005 | 0.00395274 | 5.90E-09 | 1.51E-04 | 32.36696558 |
| rs79717793 | A | G | 0.153705 | -0.0421104 | 0.00409969 | 9.50E-25 | 4.61E-04 | 99.22728334 |
| rs10824744 | G | A | 0.195895 | -0.0280181 | 0.00371985 | 5.00E-14 | 2.47E-04 | 53.18180706 |
| rs10822163 | G | C | 0.473796 | 0.0920086 | 0.00296023 | 1.00E-200 | 4.22E-03 | 911.3426266 |
| rs7921378 | C | G | 0.480441 | -0.0202981 | 0.00296474 | 7.60E-12 | 2.06E-04 | 44.23003111 |
| rs61850811 | A | G | 0.097582 | 0.0310896 | 0.00499178 | 4.70E-10 | 1.70E-04 | 36.6036054 |
| rs11188662 | T | C | 0.328895 | 0.0196989 | 0.00315077 | 4.00E-10 | 1.71E-04 | 36.83394995 |
| rs2068888 | A | G | 0.4516 | 0.0237089 | 0.0029663 | 1.30E-15 | 2.78E-04 | 59.87386839 |
| rs701810 | T | C | 0.324168 | -0.0188615 | 0.00316889 | 2.60E-09 | 1.56E-04 | 33.51747138 |
| rs2254069 | A | G | 0.119525 | -0.0325153 | 0.00455527 | 9.50E-13 | 2.23E-04 | 47.85090546 |
| rs11023881 | A | T | 0.389095 | -0.0231105 | 0.00303378 | 2.60E-14 | 2.54E-04 | 54.60098699 |
| rs10898075 | T | C | 0.168909 | -0.0250661 | 0.00395441 | 2.30E-10 | 1.76E-04 | 37.93094987 |
| rs174537 | T | G | 0.344464 | -0.0276452 | 0.00311089 | 6.30E-19 | 3.45E-04 | 74.22872109 |
| rs3015966 | C | T | 0.333462 | -0.0238625 | 0.00313563 | 2.70E-14 | 2.53E-04 | 54.43212469 |
| rs12805041 | T | C | 0.229486 | 0.0328747 | 0.00351764 | 9.10E-21 | 3.82E-04 | 82.19937985 |
| rs62618693 | T | C | 0.044458 | 0.0432177 | 0.00716904 | 1.70E-09 | 1.59E-04 | 34.12197203 |
| rs3842763 | T | G | 0.241445 | 0.0277197 | 0.00349861 | 2.30E-15 | 2.81E-04 | 60.52667135 |
| rs4938637 | A | G | 0.075177 | 0.0357712 | 0.00560887 | 1.80E-10 | 1.78E-04 | 38.25869095 |
| rs7947951 | G | A | 0.689386 | -0.028028 | 0.00319489 | 1.70E-18 | 3.36E-04 | 72.35291517 |
| rs12575636 | G | T | 0.188786 | -0.0280622 | 0.00378802 | 1.30E-13 | 2.41E-04 | 51.8675355 |
| rs1871395 | G | A | 0.152399 | -0.0533039 | 0.00411869 | 2.60E-38 | 7.34E-04 | 157.9256423 |
| rs11172134 | A | T | 0.202001 | 0.0312034 | 0.00368476 | 2.50E-17 | 3.14E-04 | 67.50535794 |
| rs7298698 | C | T | 0.468281 | -0.0559534 | 0.00296513 | 2.00E-79 | 1.56E-03 | 335.7078587 |
| rs377252458 | A | C | 0.6883 | 0.019189 | 0.00320365 | 2.10E-09 | 1.58E-04 | 33.97270089 |
| rs7304705 | G | A | 0.078492 | 0.0553875 | 0.00549749 | 7.10E-24 | 4.44E-04 | 95.45155432 |
| rs117233107 | A | G | 0.015052 | 0.1107 | 0.0128532 | 7.10E-18 | 3.63E-04 | 78.14528538 |
| rs11110390 | T | C | 0.33294 | -0.0233781 | 0.00313808 | 9.30E-14 | 2.43E-04 | 52.20317722 |
| rs141881700 | A | G | 0.013025 | -0.119796 | 0.0133069 | 2.20E-19 | 3.69E-04 | 79.35439548 |
| rs5009837 | C | T | 0.715685 | -0.0276323 | 0.00327168 | 3.00E-17 | 3.11E-04 | 66.82407007 |
| rs825453 | T | A | 0.608051 | -0.0232519 | 0.00303328 | 1.80E-14 | 2.58E-04 | 55.41669082 |
| rs11064358 | A | G | 0.13584 | -0.0262817 | 0.00432125 | 1.20E-09 | 1.62E-04 | 34.86915062 |
| rs1989390 | A | C | 0.832772 | -0.023683 | 0.00402738 | 4.10E-09 | 1.56E-04 | 33.59066383 |
| rs75130744 | C | G | 0.070756 | -0.0530844 | 0.00577968 | 4.10E-20 | 3.71E-04 | 79.69480665 |
| rs80127816 | A | G | 0.051979 | -0.0384471 | 0.00666265 | 7.90E-09 | 1.46E-04 | 31.32409414 |
| rs7310409 | G | A | 0.619636 | 0.0271474 | 0.00304268 | 4.60E-19 | 3.47E-04 | 74.71118493 |
| rs2130382 | G | C | 0.312001 | 0.0206428 | 0.00319336 | 1.00E-10 | 1.83E-04 | 39.33712858 |
| rs10841521 | G | A | 0.267714 | 0.0185794 | 0.00338289 | 4.00E-08 | 1.35E-04 | 29.10155283 |
| rs4307773 | C | T | 0.582524 | -0.0264537 | 0.00299133 | 9.30E-19 | 3.40E-04 | 73.1995186 |
| rs36179992 | G | GTA | 0.494256 | -0.0196018 | 0.00303598 | 1.10E-10 | 1.92E-04 | 41.30477145 |
| rs1239945 | A | G | 0.541295 | -0.0162516 | 0.00297052 | 4.50E-08 | 1.31E-04 | 28.20063568 |
| rs35792308 | C | CA | 0.672588 | -0.019514 | 0.00326807 | 2.40E-09 | 1.68E-04 | 36.06212767 |
| rs61755579 | T | C | 0.030406 | 0.0549996 | 0.0086004 | 1.60E-10 | 1.78E-04 | 38.35199282 |
| rs2239222 | G | A | 0.349946 | 0.019216 | 0.00312118 | 7.40E-10 | 1.68E-04 | 36.12364878 |
| rs13379043 | C | T | 0.279423 | 0.0201121 | 0.00335821 | 2.10E-09 | 1.63E-04 | 35.02436969 |
| rs17580 | A | T | 0.047472 | 0.0459238 | 0.00695892 | 4.10E-11 | 1.91E-04 | 41.01247229 |
| rs2498786 | G | C | 0.615674 | -0.0226626 | 0.00304528 | 9.90E-14 | 2.43E-04 | 52.26582509 |
| rs28929474 | T | C | 0.019899 | 0.124662 | 0.0106081 | 6.90E-32 | 6.06E-04 | 130.3994619 |
| rs34210072 | A | G | 0.188971 | 0.0237447 | 0.00378804 | 3.60E-10 | 1.73E-04 | 37.16055359 |
| rs11621792 | T | C | 0.452552 | -0.0480818 | 0.0029971 | 6.40E-58 | 1.15E-03 | 246.5544076 |
| rs144673482 | AAAAC | A | 0.840934 | -0.0223375 | 0.0040488 | 3.40E-08 | 1.33E-04 | 28.70176304 |
| rs8025155 | G | C | 0.138996 | -0.0449663 | 0.00430213 | 1.40E-25 | 4.84E-04 | 104.0960396 |
| rs79391862 | C | A | 0.013925 | -0.148247 | 0.0128269 | 6.80E-31 | 6.04E-04 | 129.8318866 |
| rs28539993 | T | C | 0.424169 | 0.0266937 | 0.00299324 | 4.70E-19 | 3.48E-04 | 74.859153 |
| rs11637595 | T | C | 0.275427 | -0.0186549 | 0.0033492 | 2.50E-08 | 1.39E-04 | 29.86599804 |
| rs12914895 | G | C | 0.485846 | 0.0166813 | 0.00297486 | 2.10E-08 | 1.39E-04 | 29.89194756 |
| rs79240050 | A | G | 0.030048 | 0.0580649 | 0.00915163 | 2.20E-10 | 1.97E-04 | 42.25914759 |
| rs56332871 | A | C | 0.274457 | 0.0749107 | 0.00332768 | 3.20E-112 | 2.23E-03 | 481.547418 |
| rs139974673 | C | T | 0.026337 | -0.0834946 | 0.00923435 | 1.50E-19 | 3.58E-04 | 76.89343229 |
| rs7194995 | A | C | 0.768827 | -0.0277241 | 0.00352084 | 3.40E-15 | 2.73E-04 | 58.75455181 |
| rs11644601 | C | T | 0.298714 | 0.0240504 | 0.00323789 | 1.10E-13 | 2.42E-04 | 52.1126081 |
| rs62033400 | G | A | 0.392517 | -0.0236057 | 0.00303462 | 7.30E-15 | 2.66E-04 | 57.14575653 |
| rs2925979 | C | T | 0.70015 | 0.0253781 | 0.00323025 | 4.00E-15 | 2.70E-04 | 58.15311852 |
| rs10083762 | G | C | 0.273859 | 0.0194611 | 0.003323 | 4.70E-09 | 1.51E-04 | 32.38846483 |
| rs12444108 | A | G | 0.401832 | -0.0177667 | 0.00301912 | 4.00E-09 | 1.52E-04 | 32.62791595 |
| rs7220103 | G | A | 0.040603 | 0.0603862 | 0.00742767 | 4.30E-16 | 2.84E-04 | 61.0938472 |
| rs72844546 | T | C | 0.653389 | -0.0213563 | 0.00307746 | 3.90E-12 | 2.07E-04 | 44.42200007 |
| rs191273168 | A | G | 0.020966 | -0.192149 | 0.0109076 | 1.90E-69 | 1.52E-03 | 326.3552303 |
| rs17880847 | A | T | 0.013246 | -0.203969 | 0.0131101 | 1.40E-54 | 1.09E-03 | 234.0651475 |
| rs80028739 | A | G | 0.009688 | 0.129658 | 0.0157991 | 2.30E-16 | 3.23E-04 | 69.37246393 |
| rs10153315 | C | T | 0.418469 | -0.0191278 | 0.0029778 | 1.30E-10 | 1.78E-04 | 38.29003331 |
| rs12947017 | T | C | 0.401504 | 0.129396 | 0.00305543 | 1.00E-200 | 8.05E-03 | 1743.989397 |
| rs117387630 | T | C | 0.021749 | -0.307189 | 0.0105124 | 1.00E-187 | 4.02E-03 | 866.7433022 |
| rs9911944 | T | G | 0.487919 | -0.0403962 | 0.00293566 | 4.40E-43 | 8.15E-04 | 175.4542551 |
| rs9907478 | T | C | 0.087112 | 0.131801 | 0.00519306 | 4.20E-142 | 2.76E-03 | 595.6301259 |
| rs142331290 | G | A | 0.015933 | -0.172662 | 0.011754 | 7.50E-49 | 9.35E-04 | 201.1704316 |
| rs140215187 | G | A | 0.028766 | -0.0598337 | 0.00990605 | 1.50E-09 | 2.00E-04 | 43.01541868 |
| rs57116414 | A | G | 0.031495 | -0.149229 | 0.00843372 | 4.60E-70 | 1.36E-03 | 292.470825 |
| rs7224815 | T | A | 0.408313 | -0.0326919 | 0.00298501 | 6.50E-28 | 5.16E-04 | 111.0790319 |
| rs12943365 | G | C | 0.610002 | 0.0215562 | 0.0030076 | 7.70E-13 | 2.21E-04 | 47.54187121 |
| rs8178824 | T | C | 0.030223 | -0.0816256 | 0.00858555 | 2.00E-21 | 3.91E-04 | 83.99899157 |
| rs8087306 | C | G | 0.686601 | -0.0189063 | 0.00319009 | 3.10E-09 | 1.54E-04 | 33.07684942 |
| rs663640 | T | C | 0.218928 | -0.022188 | 0.00357678 | 5.50E-10 | 1.68E-04 | 36.20295462 |
| rs1217569 | A | G | 0.211972 | -0.0241018 | 0.00366856 | 5.00E-11 | 1.94E-04 | 41.72973128 |
| rs4804416 | G | T | 0.42688 | -0.0361902 | 0.00298919 | 9.70E-34 | 6.41E-04 | 137.8649745 |
| rs75702986 | A | G | 0.18616 | 0.0295021 | 0.00383889 | 1.50E-14 | 2.64E-04 | 56.71370377 |
| rs1350292 | T | C | 0.933163 | 0.0640282 | 0.00591625 | 2.70E-27 | 5.11E-04 | 109.9971386 |
| rs60321073 | G | A | 0.178706 | -0.0240354 | 0.00386749 | 5.10E-10 | 1.70E-04 | 36.46333107 |
| rs8104606 | A | G | 0.649839 | 0.0175703 | 0.0031019 | 1.50E-08 | 1.40E-04 | 30.20891416 |
| rs34255979 | T | C | 0.120529 | 0.0511146 | 0.00455471 | 3.20E-29 | 5.54E-04 | 119.1477453 |
| rs941408 | T | C | 0.287351 | 0.0453878 | 0.0032662 | 6.70E-44 | 8.44E-04 | 181.541295 |
| rs202200760 | C | G | 0.039306 | 0.155205 | 0.00830682 | 6.70E-78 | 1.82E-03 | 391.8215664 |
| rs273492 | A | G | 0.255863 | -0.0279686 | 0.00338989 | 1.60E-16 | 2.98E-04 | 64.05800685 |
| rs74444983 | C | T | 0.258852 | -0.020953 | 0.00339712 | 6.90E-10 | 1.68E-04 | 36.22131819 |
| rs67611724 | T | C | 0.154399 | -0.0249406 | 0.00408629 | 1.00E-09 | 1.62E-04 | 34.92500739 |
| rs2618567 | T | G | 0.657848 | -0.0193661 | 0.00312356 | 5.60E-10 | 1.69E-04 | 36.30316642 |
| rs6129802 | T | C | 0.213305 | 0.0235482 | 0.00360894 | 6.80E-11 | 1.86E-04 | 40.01712493 |
| rs310670 | C | G | 0.650093 | -0.0173515 | 0.00312717 | 2.90E-08 | 1.37E-04 | 29.45125833 |
| rs6120663 | A | C | 0.440041 | -0.0193988 | 0.0029854 | 8.10E-11 | 1.85E-04 | 39.87694196 |
| rs55987409 | T | C | 0.072173 | 0.0322976 | 0.00573092 | 1.70E-08 | 1.40E-04 | 30.03897711 |
| rs6073431 | T | C | 0.531548 | 0.0327618 | 0.00301919 | 2.00E-27 | 5.35E-04 | 114.9787271 |
| rs6063803 | G | A | 0.581469 | -0.0194566 | 0.00300595 | 9.60E-11 | 1.84E-04 | 39.61962142 |
| rs4820091 | G | T | 0.182395 | 0.0244305 | 0.00386379 | 2.60E-10 | 1.78E-04 | 38.27724662 |
| rs757869 | A | G | 0.707611 | -0.0306421 | 0.00325743 | 5.10E-21 | 3.89E-04 | 83.56099836 |
| rs165722 | T | C | 0.515456 | -0.0172758 | 0.00298459 | 7.10E-09 | 1.49E-04 | 32.05590933 |
| rs3747207 | A | G | 0.215014 | 0.0355567 | 0.00360423 | 5.90E-23 | 4.27E-04 | 91.79079404 |
| SNP single nucleotide polymorphisms, EA efect allele, OA other allele, se standard error | | | | | | | | |
