# Supplementary material for: Examining the causal relationship between sex hormone-binding globulin (SHBG) and infertility: A Mendelian randomization study
Source: PLoS One. 2024 Jun 7;19(6):e0304216. doi: 10.1371/journal.pone.0304216 (PMC11161117; doi:10.1371/journal.pone.0304216)
Supplement: S2 Table — (DOCX) [file pone.0304216.s002.docx]

# Supplementary Table 2 Basic information of SNPs associated with sex hormone binding globulin in male

| SNP | ea | oa | eaf | beta | se | pval | r2 | f |
| --- | --- | --- | --- | --- | --- | --- | --- | --- |
| rs115276619 | A | T | 0.01654 | -0.0745279 | 0.0128 | 5.80E-09 | 1.81E-04 | 33.47526235 |
| rs35877733 | T | C | 0.845594 | 0.0239774 | 0.00439349 | 4.80E-08 | 1.50E-04 | 27.81063961 |
| rs10864086 | A | C | 0.74561 | -0.0368555 | 0.00364338 | 4.70E-24 | 5.15E-04 | 95.4895282 |
| rs4590622 | A | G | 0.288149 | 0.0247127 | 0.00354346 | 3.10E-12 | 2.51E-04 | 46.41634749 |
| rs7552212 | T | C | 0.204464 | -0.0293305 | 0.00392461 | 7.80E-14 | 2.80E-04 | 51.85047197 |
| rs28549287 | A | G | 0.774251 | -0.0322801 | 0.00411106 | 4.10E-15 | 3.64E-04 | 67.49181497 |
| rs150313019 | G | C | 0.208717 | -0.025099 | 0.00390077 | 1.20E-10 | 2.08E-04 | 38.54858179 |
| rs71586027 | TATCTC | T | 0.479544 | 0.02631 | 0.00316937 | 1.00E-16 | 3.46E-04 | 64.02060814 |
| rs2281003 | C | T | 0.4668 | -0.0178547 | 0.00317717 | 1.90E-08 | 1.59E-04 | 29.39751079 |
| rs2642438 | G | A | 0.701824 | 0.0355439 | 0.00345617 | 8.30E-25 | 5.29E-04 | 97.98876568 |
| rs2045345 | G | A | 0.195481 | -0.0295402 | 0.00408556 | 4.80E-13 | 2.74E-04 | 50.85134784 |
| rs72929847 | A | G | 0.075594 | 0.0493165 | 0.00600329 | 2.10E-16 | 3.40E-04 | 62.97928898 |
| rs1762509 | G | A | 0.655605 | -0.0612053 | 0.00334461 | 8.30E-75 | 1.69E-03 | 313.8542241 |
| rs36086195 | T | C | 0.577156 | 0.0404596 | 0.00319828 | 1.10E-36 | 7.99E-04 | 148.1082422 |
| rs35189848 | G | A | 0.446037 | -0.0276408 | 0.00318506 | 4.00E-18 | 3.78E-04 | 69.9571961 |
| rs79598313 | T | C | 0.02316 | -0.213956 | 0.0105136 | 4.60E-92 | 2.07E-03 | 384.4385615 |
| rs201468966 | C | CA | 0.766759 | 0.0364362 | 0.00377278 | 4.60E-22 | 4.75E-04 | 87.99373206 |
| rs72708162 | T | G | 0.078495 | 0.0386218 | 0.00587371 | 4.90E-11 | 2.16E-04 | 39.97734692 |
| rs72787581 | A | G | 0.023084 | 0.0707285 | 0.0106683 | 3.40E-11 | 2.26E-04 | 41.79946404 |
| rs6736913 | G | A | 0.979119 | -0.0846117 | 0.0110529 | 1.90E-14 | 2.93E-04 | 54.23633029 |
| rs6546096 | G | A | 0.737087 | -0.0356272 | 0.00361112 | 5.80E-23 | 4.92E-04 | 91.16401251 |
| rs6541725 | A | C | 0.380867 | -0.0190861 | 0.00326145 | 4.90E-09 | 1.72E-04 | 31.82598373 |
| rs13029936 | T | C | 0.182948 | 0.02599 | 0.00409027 | 2.10E-10 | 2.02E-04 | 37.41046989 |
| rs2943646 | G | A | 0.647532 | -0.0314343 | 0.00331118 | 2.20E-21 | 4.51E-04 | 83.5795402 |
| rs1260326 | C | T | 0.603649 | 0.0913154 | 0.00323084 | 9.71E-176 | 3.99E-03 | 742.000787 |
| rs72798731 | T | C | 0.033951 | 0.0633323 | 0.00896022 | 1.60E-12 | 2.63E-04 | 48.74522263 |
| rs4853180 | C | T | 0.424013 | -0.0222845 | 0.00324763 | 6.80E-12 | 2.43E-04 | 44.93849566 |
| rs17041868 | C | T | 0.064051 | -0.0386263 | 0.00645125 | 2.10E-09 | 1.79E-04 | 33.13885847 |
| rs6717858 | C | T | 0.40216 | 0.0287395 | 0.00323055 | 5.80E-19 | 3.97E-04 | 73.59194985 |
| rs9288177 | A | G | 0.373539 | -0.0202455 | 0.00329043 | 7.60E-10 | 1.92E-04 | 35.53741669 |
| rs11454712 | C | CA | 0.601157 | 0.026825 | 0.00345601 | 8.40E-15 | 3.45E-04 | 63.93442445 |
| rs9811216 | C | T | 0.261317 | 0.0203838 | 0.0036094 | 1.60E-08 | 1.60E-04 | 29.71537859 |
| rs6792725 | G | A | 0.69378 | 0.0259319 | 0.00354169 | 2.40E-13 | 2.86E-04 | 52.93752631 |
| rs511154 | G | A | 0.765441 | -0.0645704 | 0.00373704 | 6.80E-67 | 1.50E-03 | 277.7134995 |
| rs4135250 | G | A | 0.120985 | 0.0393153 | 0.00485016 | 5.20E-16 | 3.29E-04 | 60.91290844 |
| rs3729931 | A | G | 0.357554 | 0.0198673 | 0.00329209 | 1.60E-09 | 1.81E-04 | 33.59311382 |
| rs79287178 | A | G | 0.031519 | -0.090053 | 0.00952221 | 3.20E-21 | 4.95E-04 | 91.74671171 |
| rs7696472 | A | G | 0.475333 | 0.0501085 | 0.00317075 | 3.00E-56 | 1.25E-03 | 232.2545824 |
| rs28507491 | A | G | 0.375258 | 0.032595 | 0.00326965 | 2.10E-23 | 4.98E-04 | 92.31336107 |
| rs58833496 | T | A | 0.146448 | -0.0254415 | 0.00448742 | 1.40E-08 | 1.62E-04 | 29.9767585 |
| rs2073503 | G | C | 0.310192 | 0.0187904 | 0.00342339 | 4.00E-08 | 1.51E-04 | 27.99058835 |
| rs70957254 | AC | A | 0.132068 | 0.0288182 | 0.00470804 | 9.30E-10 | 1.90E-04 | 35.27079336 |
| rs34190889 | G | A | 0.110578 | 0.0336043 | 0.00507903 | 3.70E-11 | 2.22E-04 | 41.15080293 |
| rs73193388 | T | C | 0.136139 | 0.026423 | 0.00462767 | 1.10E-08 | 1.64E-04 | 30.4212832 |
| rs4860987 | T | A | 0.259115 | 0.0729585 | 0.00381721 | 2.00E-81 | 2.04E-03 | 379.3142625 |
| rs3775327 | C | T | 0.751012 | -0.0319026 | 0.00368547 | 4.90E-18 | 3.81E-04 | 70.52747244 |
| rs78890745 | A | G | 0.107909 | 0.035134 | 0.00512956 | 7.40E-12 | 2.38E-04 | 44.02919815 |
| rs13108218 | G | A | 0.614877 | -0.0565755 | 0.00327979 | 1.10E-66 | 1.52E-03 | 281.2023133 |
| rs11734408 | G | A | 0.290983 | 0.020603 | 0.00348705 | 3.50E-09 | 1.75E-04 | 32.44716786 |
| rs13150068 | G | A | 0.436463 | -0.048378 | 0.0031939 | 7.90E-52 | 1.15E-03 | 213.4919578 |
| rs6532796 | G | A | 0.706224 | -0.0497219 | 0.0034742 | 1.80E-46 | 1.03E-03 | 190.2021834 |
| rs72666817 | A | G | 0.204068 | 0.0261021 | 0.00393817 | 3.40E-11 | 2.21E-04 | 41.00279237 |
| rs111366116 | T | C | 0.112719 | -0.0396388 | 0.00501926 | 2.80E-15 | 3.14E-04 | 58.23061168 |
| rs34576922 | G | C | 0.247562 | 0.0234211 | 0.00368287 | 2.00E-10 | 2.04E-04 | 37.85938655 |
| rs11743810 | T | C | 0.559153 | 0.0222265 | 0.00319528 | 3.50E-12 | 2.44E-04 | 45.12134382 |
| rs138611541 | C | T | 0.077113 | -0.0381385 | 0.00593873 | 1.30E-10 | 2.07E-04 | 38.35383503 |
| rs11739158 | T | C | 0.427543 | 0.0191199 | 0.00321034 | 2.60E-09 | 1.79E-04 | 33.15027664 |
| rs34502855 | A | G | 0.426799 | 0.0182912 | 0.0032219 | 1.40E-08 | 1.64E-04 | 30.3250384 |
| rs10041660 | C | T | 0.505425 | 0.0192845 | 0.00320712 | 1.80E-09 | 1.86E-04 | 34.44307599 |
| rs40270 | C | A | 0.771541 | -0.0331526 | 0.0037746 | 1.60E-18 | 3.87E-04 | 71.79369502 |
| rs9379084 | A | G | 0.115528 | -0.0346877 | 0.00510523 | 1.10E-11 | 2.46E-04 | 45.55590601 |
| rs4715316 | T | C | 0.643873 | 0.0202711 | 0.00332066 | 1.00E-09 | 1.88E-04 | 34.91058508 |
| rs9379802 | A | T | 0.402503 | 0.0275041 | 0.00322931 | 1.60E-17 | 3.64E-04 | 67.41760306 |
| rs6939861 | A | G | 0.261697 | -0.0268266 | 0.00364951 | 2.00E-13 | 2.78E-04 | 51.52297927 |
| rs2665357 | C | A | 0.516901 | -0.0362209 | 0.00317707 | 4.10E-30 | 6.55E-04 | 121.4401153 |
| rs35694679 | ACTCT | A | 0.960551 | -0.0539542 | 0.00823662 | 5.70E-11 | 2.21E-04 | 40.87128533 |
| rs3132469 | G | A | 0.850932 | -0.0394517 | 0.0044473 | 7.30E-19 | 3.95E-04 | 73.16405606 |
| rs7744830 | G | T | 0.689114 | -0.0229461 | 0.00342656 | 2.10E-11 | 2.26E-04 | 41.79492218 |
| rs1229492 | C | T | 0.730031 | -0.0264419 | 0.00359122 | 1.80E-13 | 2.76E-04 | 51.05940546 |
| rs10278546 | C | A | 0.193559 | 0.0363244 | 0.00402532 | 1.80E-19 | 4.12E-04 | 76.32684838 |
| rs2694157 | C | T | 0.620165 | 0.0217961 | 0.00326107 | 2.30E-11 | 2.24E-04 | 41.46413023 |
| rs39749 | T | C | 0.42614 | -0.0211431 | 0.00322853 | 5.80E-11 | 2.19E-04 | 40.50476135 |
| rs157935 | G | T | 0.302777 | 0.0300095 | 0.00346114 | 4.30E-18 | 3.80E-04 | 70.4519143 |
| rs62472728 | T | C | 0.061521 | 0.0388978 | 0.00666919 | 5.50E-09 | 1.75E-04 | 32.36603042 |
| rs35368205 | T | C | 0.19791 | 0.0223507 | 0.00397007 | 1.80E-08 | 1.59E-04 | 29.38038717 |
| rs11765639 | A | G | 0.386532 | -0.0208357 | 0.0032643 | 1.70E-10 | 2.06E-04 | 38.14156541 |
| rs2302479 | G | A | 0.576025 | 0.0209232 | 0.00321353 | 7.50E-11 | 2.14E-04 | 39.61377221 |
| rs115946508 | A | C | 0.111157 | 0.0448585 | 0.0050719 | 9.20E-19 | 3.98E-04 | 73.67830444 |
| rs849133 | T | C | 0.502255 | 0.023737 | 0.00316888 | 6.90E-14 | 2.82E-04 | 52.19401796 |
| rs7794048 | A | G | 0.06373 | -0.035812 | 0.00651402 | 3.80E-08 | 1.53E-04 | 28.35203149 |
| rs6950023 | G | T | 0.814859 | 0.0776829 | 0.00407488 | 5.00E-81 | 1.82E-03 | 337.8648044 |
| rs12543287 | C | G | 0.370996 | 0.0265496 | 0.00330275 | 9.10E-16 | 3.29E-04 | 60.9532725 |
| rs150539196 | G | A | 0.035697 | 0.0894658 | 0.00878756 | 2.40E-24 | 5.51E-04 | 102.1208099 |
| rs1991323 | T | C | 0.103508 | 0.0337064 | 0.00524433 | 1.30E-10 | 2.11E-04 | 39.06179423 |
| rs876435 | A | G | 0.590766 | -0.0186658 | 0.00325624 | 9.90E-09 | 1.68E-04 | 31.20822857 |
| rs9297994 | A | G | 0.663546 | 0.030556 | 0.00335425 | 8.30E-20 | 4.17E-04 | 77.24784184 |
| rs7822342 | G | A | 0.588278 | 0.0203078 | 0.00322677 | 3.10E-10 | 2.00E-04 | 37.00962948 |
| rs1461729 | G | A | 0.899058 | 0.0519417 | 0.00526956 | 6.40E-23 | 4.90E-04 | 90.7444739 |
| rs9693586 | C | A | 0.660886 | -0.0302364 | 0.00343252 | 1.30E-18 | 4.10E-04 | 75.93219824 |
| rs7828742 | G | A | 0.601318 | -0.0178056 | 0.00325475 | 4.50E-08 | 1.52E-04 | 28.15954358 |
| rs2721195 | C | T | 0.525212 | -0.0281438 | 0.00318673 | 1.00E-18 | 3.95E-04 | 73.19593618 |
| rs2791756 | C | A | 0.436904 | 0.0213596 | 0.00320188 | 2.50E-11 | 2.24E-04 | 41.58797055 |
| rs7860122 | G | A | 0.249915 | 0.0204873 | 0.00367223 | 2.40E-08 | 1.57E-04 | 29.15117979 |
| rs10881582 | A | G | 0.239151 | 0.0314706 | 0.00373037 | 3.30E-17 | 3.60E-04 | 66.7809833 |
| rs10868080 | A | T | 0.743399 | -0.0532888 | 0.00362005 | 4.80E-49 | 1.08E-03 | 200.8808288 |
| rs7860634 | A | G | 0.572759 | 0.0289016 | 0.00320618 | 2.00E-19 | 4.09E-04 | 75.74984125 |
| rs4979372 | C | T | 0.489099 | 0.0306061 | 0.00318601 | 7.50E-22 | 4.68E-04 | 86.74978441 |
| rs1616572 | T | C | 0.161167 | -0.0240918 | 0.00432117 | 2.50E-08 | 1.57E-04 | 29.07192703 |
| rs78679318 | G | T | 0.187391 | 0.0240626 | 0.00407771 | 3.60E-09 | 1.76E-04 | 32.66687422 |
| rs9697210 | A | G | 0.145266 | -0.0443715 | 0.0045101 | 7.70E-23 | 4.89E-04 | 90.60060435 |
| rs68040629 | G | C | 0.534879 | -0.0176832 | 0.00317573 | 2.60E-08 | 1.56E-04 | 28.8221464 |
| rs4918722 | T | C | 0.725454 | -0.0232926 | 0.0035396 | 4.70E-11 | 2.16E-04 | 40.03781213 |
| rs3006593 | C | G | 0.379451 | 0.0182367 | 0.00325436 | 2.10E-08 | 1.57E-04 | 29.01401598 |
| rs79717793 | A | G | 0.153928 | -0.0579424 | 0.00438195 | 6.50E-40 | 8.74E-04 | 162.1113365 |
| rs1892501 | G | A | 0.195656 | -0.0388246 | 0.00397964 | 1.70E-22 | 4.74E-04 | 87.91654293 |
| rs7922024 | T | C | 0.049055 | 0.0421167 | 0.00735585 | 1.00E-08 | 1.65E-04 | 30.65736037 |
| rs72824931 | T | C | 0.061059 | -0.0466521 | 0.00661577 | 1.80E-12 | 2.50E-04 | 46.23323788 |
| rs1772183 | A | G | 0.478523 | -0.030415 | 0.00316265 | 6.80E-22 | 4.62E-04 | 85.55190808 |
| rs1408579 | T | C | 0.496935 | 0.0290749 | 0.00314925 | 2.60E-20 | 4.23E-04 | 78.31758306 |
| rs35095528 | C | CT | 0.148621 | 0.0429146 | 0.00513772 | 6.70E-17 | 4.66E-04 | 86.36369929 |
| rs10995445 | T | A | 0.471631 | 0.135788 | 0.00318023 | 1.00E-200 | 9.19E-03 | 1717.858514 |
| rs71485762 | T | C | 0.503386 | 0.0199456 | 0.00315797 | 2.70E-10 | 1.99E-04 | 36.84819546 |
| rs2924545 | A | G | 0.429979 | -0.0184394 | 0.00321423 | 9.60E-09 | 1.67E-04 | 30.8758977 |
| rs631695 | G | T | 0.582079 | -0.0392864 | 0.00320898 | 1.80E-34 | 7.51E-04 | 139.188183 |
| rs11564722 | T | C | 0.241228 | 0.0227435 | 0.00375723 | 1.40E-09 | 1.89E-04 | 35.07933779 |
| rs7947951 | G | A | 0.689182 | -0.030322 | 0.00342253 | 8.00E-19 | 3.94E-04 | 72.98647807 |
| rs3867138 | T | C | 0.231495 | 0.0214728 | 0.00377182 | 1.20E-08 | 1.64E-04 | 30.39152246 |
| rs77643000 | C | T | 0.128513 | 0.0264503 | 0.00480039 | 3.60E-08 | 1.57E-04 | 29.03038357 |
| rs11023881 | A | T | 0.388973 | -0.0257382 | 0.00324973 | 2.40E-15 | 3.15E-04 | 58.34296682 |
| rs10895277 | G | A | 0.34066 | -0.0227191 | 0.00335514 | 1.30E-11 | 2.32E-04 | 42.95651634 |
| rs12797706 | A | G | 0.234894 | 0.0312567 | 0.00377669 | 1.30E-16 | 3.51E-04 | 65.06506174 |
| rs117233107 | A | G | 0.01533 | 0.108995 | 0.013678 | 1.60E-15 | 3.59E-04 | 66.45341944 |
| rs4149056 | C | T | 0.151541 | -0.0774044 | 0.00440743 | 4.80E-69 | 1.54E-03 | 285.8100379 |
| rs12320328 | G | A | 0.084393 | -0.059398 | 0.00571811 | 2.80E-25 | 5.45E-04 | 101.0442407 |
| rs11829410 | G | A | 0.053877 | -0.0439351 | 0.00700638 | 3.60E-10 | 1.97E-04 | 36.45652318 |
| rs7306330 | A | T | 0.44316 | 0.0191987 | 0.00320797 | 2.20E-09 | 1.82E-04 | 33.69994291 |
| rs540730 | C | T | 0.756166 | -0.0436693 | 0.00368377 | 2.00E-32 | 7.03E-04 | 130.3421603 |
| rs11610256 | T | C | 0.216936 | 0.0211943 | 0.00385374 | 3.80E-08 | 1.53E-04 | 28.27150036 |
| rs9738226 | G | A | 0.620364 | 0.0492166 | 0.00327096 | 3.60E-51 | 1.14E-03 | 211.5672863 |
| rs11045806 | A | G | 0.189969 | 0.034338 | 0.0040678 | 3.10E-17 | 3.63E-04 | 67.23687581 |
| rs7306128 | G | A | 0.053877 | -0.043938 | 0.00700648 | 3.60E-10 | 1.97E-04 | 36.46133702 |
| rs7314285 | G | T | 0.068885 | 0.0724648 | 0.00626139 | 5.60E-31 | 6.74E-04 | 124.8505209 |
| rs9739640 | G | A | 0.167072 | -0.025073 | 0.00431444 | 6.20E-09 | 1.75E-04 | 32.41270367 |
| rs142051220 | A | T | 0.009383 | -0.13799 | 0.0175068 | 3.20E-15 | 3.54E-04 | 65.58613063 |
| rs2298058 | T | C | 0.307329 | 0.028089 | 0.00344243 | 3.40E-16 | 3.36E-04 | 62.23927815 |
| rs62637665 | G | C | 0.196063 | 0.0238742 | 0.00399394 | 2.30E-09 | 1.80E-04 | 33.28655796 |
| rs58939796 | T | G | 0.30098 | 0.0278056 | 0.00345975 | 9.20E-16 | 3.25E-04 | 60.27658281 |
| rs28929474 | T | C | 0.019531 | 0.344615 | 0.0114655 | 1.80E-198 | 4.55E-03 | 846.2955821 |
| rs13379043 | C | T | 0.281518 | 0.0203851 | 0.00359668 | 1.40E-08 | 1.68E-04 | 31.14126834 |
| rs17580 | A | T | 0.047832 | 0.0725345 | 0.00743486 | 1.70E-22 | 4.79E-04 | 88.80656596 |
| rs11621792 | T | C | 0.453741 | -0.028573 | 0.00320112 | 4.40E-19 | 4.05E-04 | 74.99108601 |
| rs2239222 | G | A | 0.350617 | 0.0267173 | 0.0033486 | 1.50E-15 | 3.25E-04 | 60.22485173 |
| rs55869502 | T | C | 0.495399 | -0.0264543 | 0.00317995 | 8.90E-17 | 3.50E-04 | 64.82809974 |
| rs6576009 | C | T | 0.57671 | -0.018855 | 0.00326893 | 8.00E-09 | 1.74E-04 | 32.15433133 |
| rs191093518 | A | G | 0.191667 | 0.0301786 | 0.0040313 | 7.10E-14 | 2.82E-04 | 52.28461813 |
| rs72683923 | C | T | 0.01938 | 0.0897608 | 0.011514 | 6.40E-15 | 3.06E-04 | 56.73832319 |
| rs11158976 | T | C | 0.287756 | 0.0195088 | 0.00351729 | 2.90E-08 | 1.56E-04 | 28.89998313 |
| rs1899727 | A | G | 0.13876 | -0.0466455 | 0.00459299 | 3.10E-24 | 5.20E-04 | 96.37167992 |
| rs149624078 | T | C | 0.013564 | -0.231275 | 0.0141043 | 2.00E-60 | 1.43E-03 | 265.492144 |
| rs11856926 | A | G | 0.448522 | -0.0264272 | 0.00320819 | 1.80E-16 | 3.45E-04 | 64.01471981 |
| rs56332871 | A | C | 0.274324 | 0.0716063 | 0.0035702 | 1.80E-89 | 2.04E-03 | 378.8890552 |
| rs374836079 | T | A | 0.243515 | 0.0264598 | 0.00375897 | 1.90E-12 | 2.58E-04 | 47.78886579 |
| rs55707100 | T | C | 0.025969 | -0.173481 | 0.00996273 | 6.60E-68 | 1.52E-03 | 282.4288738 |
| rs62011286 | A | G | 0.342562 | -0.0287125 | 0.00334238 | 8.70E-18 | 3.71E-04 | 68.80386486 |
| rs42945 | G | A | 0.513876 | -0.0210752 | 0.00319356 | 4.10E-11 | 2.22E-04 | 41.11125392 |
| rs12711486 | A | G | 0.66196 | -0.0299043 | 0.00336216 | 5.90E-19 | 4.00E-04 | 74.15774246 |
| rs34835 | G | A | 0.417512 | -0.0181026 | 0.00321957 | 1.90E-08 | 1.59E-04 | 29.52722376 |
| rs118064296 | C | T | 0.039244 | -0.0459538 | 0.00820566 | 2.10E-08 | 1.59E-04 | 29.49945051 |
| rs4782568 | G | C | 0.45 | 0.0363691 | 0.0032057 | 7.80E-30 | 6.55E-04 | 121.3501412 |
| rs2896906 | C | T | 0.193825 | -0.0253255 | 0.00401762 | 2.90E-10 | 2.00E-04 | 37.13280572 |
| rs11075253 | A | C | 0.296791 | 0.0227939 | 0.00348246 | 5.90E-11 | 2.17E-04 | 40.17742988 |
| rs55729432 | G | C | 0.266259 | 0.0241761 | 0.00361845 | 2.40E-11 | 2.28E-04 | 42.30914947 |
| rs8077638 | T | C | 0.219616 | 0.0468405 | 0.00377831 | 2.70E-35 | 7.52E-04 | 139.3983529 |
| rs7214410 | G | A | 0.55034 | -0.0303225 | 0.0031481 | 5.90E-22 | 4.55E-04 | 84.32542796 |
| rs11655704 | C | T | 0.315415 | 0.078472 | 0.00335877 | 1.00E-120 | 2.66E-03 | 493.8683085 |
| rs9303434 | G | A | 0.14665 | -0.0251122 | 0.00444611 | 1.60E-08 | 1.58E-04 | 29.23902369 |
| rs1801689 | C | A | 0.030469 | -0.0844628 | 0.00909797 | 1.60E-20 | 4.21E-04 | 78.09971824 |
| rs35721044 | T | C | 0.228727 | 0.155685 | 0.00397198 | 1.00E-200 | 8.55E-03 | 1597.587057 |
| rs55894190 | T | C | 0.601156 | 0.129527 | 0.00320044 | 1.00E-200 | 8.05E-03 | 1502.22347 |
| rs62053897 | T | A | 0.427109 | -0.0200652 | 0.00319177 | 3.20E-10 | 1.97E-04 | 36.50050018 |
| rs935129 | G | A | 0.672877 | -0.0205639 | 0.00336898 | 1.00E-09 | 1.86E-04 | 34.4868968 |
| rs7504026 | G | A | 0.514937 | 0.0172133 | 0.00313757 | 4.10E-08 | 1.48E-04 | 27.41955114 |
| rs36013981 | G | A | 0.394558 | -0.0176488 | 0.00320825 | 3.80E-08 | 1.49E-04 | 27.56728057 |
| rs2269856 | T | C | 0.706654 | 0.0351691 | 0.00343007 | 1.10E-24 | 5.13E-04 | 95.027169 |
| rs390200 | G | A | 0.599721 | 0.018758 | 0.00319513 | 4.30E-09 | 1.69E-04 | 31.29493324 |
| rs7212309 | G | A | 0.828808 | -0.0318793 | 0.00415746 | 1.70E-14 | 2.88E-04 | 53.43130111 |
| rs4297769 | A | G | 0.38805 | -0.0339221 | 0.00326022 | 2.40E-25 | 5.47E-04 | 101.2796121 |
| rs12950562 | T | C | 0.611317 | 0.0255282 | 0.0032144 | 2.00E-15 | 3.10E-04 | 57.37893148 |
| rs3744010 | A | G | 0.245874 | 0.0229293 | 0.00362854 | 2.60E-10 | 1.95E-04 | 36.11921703 |
| rs4092465 | G | A | 0.645793 | 0.0189967 | 0.00334352 | 1.30E-08 | 1.65E-04 | 30.58398264 |
| rs12605524 | G | A | 0.287924 | -0.021653 | 0.00353358 | 8.90E-10 | 1.92E-04 | 35.61552906 |
| rs663129 | A | G | 0.233686 | -0.024496 | 0.00375928 | 7.20E-11 | 2.15E-04 | 39.81426265 |
| rs11671010 | C | T | 0.179024 | 0.0473728 | 0.00413311 | 2.10E-30 | 6.60E-04 | 122.2649419 |
| rs111981233 | G | T | 0.079176 | 0.0600074 | 0.00585121 | 1.10E-24 | 5.25E-04 | 97.30237239 |
| rs8113367 | G | A | 0.632874 | 0.0195589 | 0.0032919 | 2.80E-09 | 1.78E-04 | 32.93168784 |
| rs4804101 | T | G | 0.439855 | -0.0301168 | 0.00320222 | 5.20E-21 | 4.47E-04 | 82.82040084 |
| rs34858588 | G | C | 0.078264 | -0.0394118 | 0.00590792 | 2.50E-11 | 2.24E-04 | 41.51776823 |
| rs34255979 | T | C | 0.119161 | 0.0657433 | 0.00490951 | 6.80E-41 | 9.07E-04 | 168.2067542 |
| rs1736180 | A | G | 0.287683 | 0.0396983 | 0.00350074 | 8.30E-30 | 6.46E-04 | 119.7091802 |
| rs60018147 | G | A | 0.120926 | 0.0298079 | 0.00510195 | 5.10E-09 | 1.89E-04 | 34.99495681 |
| rs202200760 | C | G | 0.038762 | 0.173273 | 0.00895292 | 1.90E-83 | 2.24E-03 | 415.3241402 |
| rs6088461 | T | G | 0.425211 | -0.0205424 | 0.00322339 | 1.90E-10 | 2.06E-04 | 38.21381758 |
| rs4599176 | T | G | 0.262325 | 0.0238663 | 0.00362077 | 4.40E-11 | 2.20E-04 | 40.84004558 |
| rs34587839 | A | G | 0.155552 | -0.0249521 | 0.0043897 | 1.30E-08 | 1.64E-04 | 30.30046461 |
| rs6072263 | C | T | 0.47733 | 0.0227177 | 0.00318367 | 9.60E-13 | 2.58E-04 | 47.70922999 |
| rs3212199 | G | T | 0.589381 | 0.0435518 | 0.0032857 | 4.20E-40 | 9.18E-04 | 170.2008869 |
| rs2257437 | A | G | 0.244233 | 0.0209604 | 0.00368904 | 1.30E-08 | 1.62E-04 | 30.04535749 |
| rs2330649 | A | G | 0.564848 | -0.0238447 | 0.00325744 | 2.50E-13 | 2.80E-04 | 51.78371921 |
| rs738409 | G | C | 0.216787 | 0.0718376 | 0.00384397 | 6.20E-78 | 1.75E-03 | 325.1576918 |
| rs695272 | C | T | 0.663642 | -0.040083 | 0.00335226 | 6.00E-33 | 7.17E-04 | 132.9484302 |
| SNP single nucleotide polymorphisms, EA efect allele, OA other allele, se standard error | | | | | | | | |
